# Differentiation of Hepatocellular Carcinoma from Intrahepatic Cholangiocarcinoma through MRI Radiomics

**DOI:** 10.3390/cancers15225373

**Published:** 2023-11-11

**Authors:** Ning Liu, Yaokun Wu, Yunyun Tao, Jing Zheng, Xiaohua Huang, Lin Yang, Xiaoming Zhang

**Affiliations:** 1 Medical Imaging Key Laboratory of Sichuan Province, Interventional Medical Center, Department of Radiology, Medical Research Center, Affiliated Hospital of North Sichuan Medical College, Nanchong 637000, China; 15082765186@163.com (N.L.); yaokunwu777@163.com (Y.W.); yunyuntao@163.com (Y.T.); 15082793282@163.com (J.Z.); harold1966@126.com (X.H.); zhangxm@nsmc.edu.cn (X.Z.); 2 Hospital of Chengdu Office of People’s Government of Tibetan Autonomous Region (Hospital. C.T.), Chengdu 610041, China

**Keywords:** radiomics, hepatocellular carcinoma, intrahepatic cholangiocarcinoma, differentiation

## Abstract

**Simple Summary:**

The noninvasive differentiation of hepatocellular carcinoma (HCC) from intrahepatic cholangiocarcinoma (ICC) remains challenging. In recent years, the number of studies on the application of radiomics in liver cancer has grown dramatically. However, there have been very few studies on the differentiation of HCC from ICC based on multisequence magnetic resonance imaging (MRI) radiomics. This study aimed to investigate the efficacy of a radiomics model based on pretherapeutic fat suppression T_2_-weighted imaging (FS-T_2_WI) and dynamic-contrast-enhanced MRI (DCE-MRI) features obtained from the arterial phase (AP) and portal venous phase (PVP) for noninvasively differentiating HCC from ICC.

**Abstract:**

The purpose of this study was to investigate the efficacy of magnetic resonance imaging (MRI) radiomics in differentiating hepatocellular carcinoma (HCC) from intrahepatic cholangiocarcinoma (ICC). The clinical and MRI data of 129 pathologically confirmed HCC patients and 48 ICC patients treated at the Affiliated Hospital of North Sichuan Medical College between April 2016 and December 2021 were retrospectively analyzed. The patients were randomly divided at a ratio of 7:3 into a training group of 124 patients (90 with HCC and 34 with ICC) and a validation group of 53 patients (39 with HCC and 14 with ICC). Radiomic features were extracted from axial fat suppression T_2_-weighted imaging (FS-T_2_WI) and axial arterial-phase (AP) and portal-venous-phase (PVP) dynamic-contrast-enhanced MRI (DCE-MRI) sequences, and the corresponding datasets were generated. The least absolute shrinkage and selection operator (LASSO) method was used to select the best radiomic features. Logistic regression was used to establish radiomic models for each sequence (FS-T_2_WI, AP and PVP models), a clinical model for optimal clinical variables (C model) and a joint radiomics model (JR model) integrating the radiomics features of all the sequences as well as a radiomics–clinical model combining optimal radiomic features and clinical risk factors (RC model). The performance of each model was evaluated using the area under the receiver operating characteristic curve (AUC). The AUCs of the FS-T_2_WI, AP, PVP, JR, C and RC models for distinguishing HCC from ICC were 0.693, 0.863, 0.818, 0.914, 0.936 and 0.977 in the training group and 0.690, 0.784, 0.727, 0.802, 0.860 and 0.877 in the validation group, respectively. The results of this study suggest that MRI-based radiomics may help noninvasively differentiate HCC from ICC. The model integrating the radiomics features and clinical risk factors showed a further improvement in performance.

## 1. Introduction

Hepatocellular carcinoma (HCC) and intrahepatic cholangiocarcinoma (ICC) are the most common types of primary liver cancer, with the former accounting for approximately 75–85% of cases [1,2,3], and their morbidity rates are increasing [4,5,6,7,8]. The treatment strategies for and prognosis of patients with HCC and ICC are very different [2,3,9,10,11,12,13,14,15,16,17,18,19]. If resection is considered feasible, resection is the treatment of choice for both entities. However, only a minority of patients are candidates for curative-intent resection. In non-resectable cases, HCC mainly responds to transcatheter arterial chemoembolization, targeted therapy and immunotherapy, while ICC benefits from classical chemotherapy, targeted therapy and immunotherapy [17,20]. Therefore, accurate pretherapeutic differentiation between HCC and ICC is essential.

At present, the noninvasive differentiation of HCC from ICC remains challenging. For example, the sensitivity and specificity of various serum tumor markers, including alpha-fetoprotein (AFP) and carbohydrate antigen 19-9 (CA19-9), are unsatisfactory [21,22,23,24]. The presentation of HCC and ICC on dynamic-contrast-enhanced computed tomography (CT) and magnetic resonance imaging (MRI) is mostly typical [25,26,27,28]. However, both HCC and ICC may occur in patients with chronic hepatitis, and imaging enhancement patterns tend to be similar in some patients with both HCC and ICC [3,29,30,31,32,33]. In addition, the enhancement may be unremarkable or atypical in some HCC cases (especially in cases of small, hypovascular or sclerosing HCC lesions) [34,35,36]. Traditional medical imaging analysis relies heavily on the physician’s subjective judgment and is thus prone to misdiagnosis [37]. Liver biopsy remains the gold standard for the final diagnosis, but this invasive procedure is refused by some patients [38]. Therefore, a pretherapeutic noninvasive method for distinguishing HCC from ICC is urgently needed.

Based on existing medical imaging modalities such as CT and MRI, an emerging technique called radiomics [39] can be used to convert intrinsic pathophysiological information that is invisible to the human eye into high-dimensional quantitative image features, which can then be used to perform tumor classification via an analysis of the relationship between these features and clinical/genetic data [39,40,41]. Studies have shown that radiomics exhibits unique advantages in classifying the disease and predicting the prognosis of patients with liver cancer [39,42,43,44,45,46,47,48,49,50,51,52,53,54,55,56]. However, there have been very few studies on the differentiation of HCC from ICC based on multisequence MRI radiomics to date. In this paper, the efficacy of a radiomic model based on pretherapeutic fat suppression T_2_-weighted imaging (FS-T_2_WI) and dynamic-contrast-enhanced MRI (DCE-MRI) features in the arterial phase (AP) and portal venous phase (PVP) for noninvasively differentiating HCC from ICC was investigated.

## 2. Materials and Methods

### 2.1. Patients

**Figure 1:**
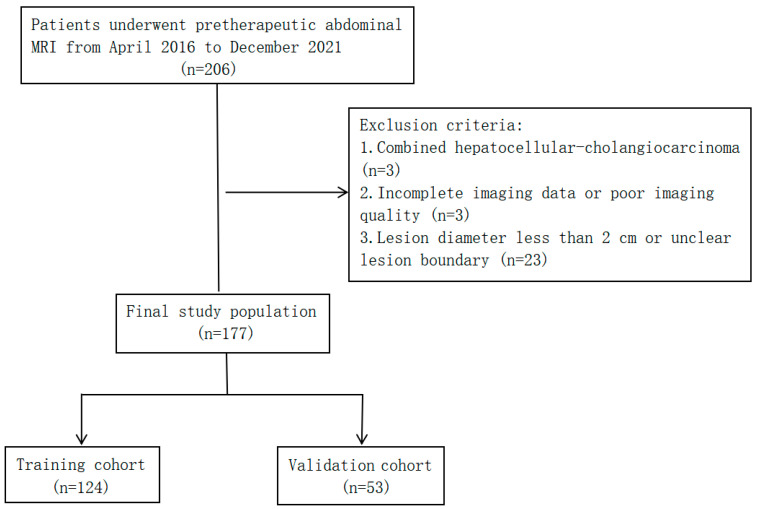
Flow chart of study population selection.

The pretherapeutic MRI and clinical data of HCC and ICC patients treated at the Affiliated Hospital of North Sichuan Medical College between April 2016 and December 2021 were retrospectively analyzed. The inclusion criteria were as follows: (1) a pathological diagnosis of HCC or ICC; (2) multisequence MRI of the upper abdomen performed within 4 weeks prior to treatment; and (3) no antitumor-related treatment prior to the MRI scan. The exclusion criteria were as follows: (1) combined hepatocellular cholangiocarcinoma (cHCC-CC); (2) incomplete data or poor MR image quality; and (3) lesion diameter <2 cm or unclear lesion contours. The data of 206 patients with primary liver cancer (145 with HCC and 61 with ICC) were collected, and 177 (129 with HCC and 48 with ICC) met the inclusion and exclusion criteria and were finally enrolled in this study. The patients were randomly divided at a 7:3 ratio into a training group (*n* = 124, 90 with HCC and 34 with ICC) and a validation group (*n* = 53, 39 with HCC and 14 with ICC) (Figure 1).

The following clinical data were acquired: age; sex; cirrhosis status; hepatitis B serological test results; pseudocapsule status; hemorrhagic necrosis status; extrahepatic metastasis status; portal vein tumor thrombus status; number of tumors; ascites status; maximum tumor diameter; abnormal prothrombin; AFP level; carcinoembryonic antigen (CEA) level; and CA19-9 level. The levels of tumor markers were measured within one week before treatment.

### 2.2. MRI Acquisition

MRI scans were performed using a Discovery 750 3.0 T superconductivity MRI scanner with a 32-channel phased-array surface coil (GE, USA). Prior to the MRI scans, all subjects fasted for 4 h and received training in breathing exercises. Scan sequences included axial 3D liver acceleration volume acquisition (LAVA), FS-T_2_WI and axial 3D LAVA dynamic-contrast-enhanced sequences (Table 1). Gd-DTPA at a dose of 15–20 mL was used as the contrast agent for dynamic contrast enhancement and injected into the dorsal vein of the hand at 2–2.5 mL/s using a high-pressure syringe. DCE MR images were obtained in the AP (18–25 s) and PVP (45–60 s) after injection of the contrast agent.

### 2.3. Image Segmentation and Feature Extraction

**Figure 2:**
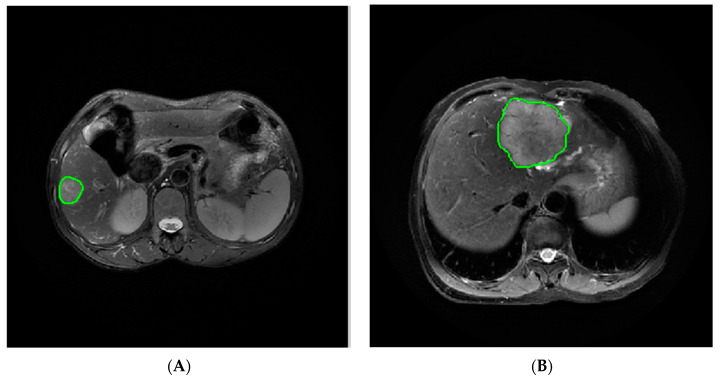
Delineation of the ROI along the edge of the lesion: (**A**) ROI segmentation on FS-T_2_WI in the case of HCC and (**B**) ICC, respectively.

The MR images of the patients in the FS-T_2_WI and AP as well as PVP were exported in digital imaging and communications in medicine (DICOM) format and imported into IBEX software (β1.0, https://sourceforge.net/projects/ibex-mda/, accessed on 13 November 2021) for tumor image segmentation. Without knowing the pathological results, an observer with 6 years of experience in abdominal radiology used the IBEX software to delineate the region of interest (ROI) along the edge of the lesion that contained the tumor layer by layer, and the entire tumor volume was manually delineated (Figure 2). After segmenting the MR images, the gray-level run-length matrix (GLRLM), gray-level cooccurrence matrix (GLCM), intensity histogram and shape features were extracted and used to construct the FS-T_2_WI, AP and PVP datasets.

### 2.4. Feature Selection

Altogether, 61 patients (42 with HCC and 19 with ICC) were randomly selected for intra- and intergroup consistency analysis. Interobserver consistency was assessed by comparing the segmentation results of two radiologists (observers 1 and 2, with 5 and 6 years of experience, respectively). Intraobserver consistency was assessed by comparing the segmentation results obtained by observer 2 more than one week after the initial results were obtained. The intraclass correlation coefficient was used to assess interobserver consistency, with a coefficient ≥0.75 being considered to indicate good consistency. To eliminate discrepancies in the index dimension, all data were standardized via Z-score normalization. The dataset generated by each sequence was subjected to intra- and interobserver consistency tests. Features with an intraclass correlation coefficient <0.75 were eliminated.

From the stable features that remained, features that significantly differentiated HCC from ICC were selected using one-way statistical analysis (independent-samples *t* test or Mann–Whitney U test, according to the characteristics of the data distribution) (*p* < 0.05). To avoid overfitting, least absolute shrinkage and selection operator (LASSO) regression analysis was performed to select the core radiomics features in differentiating HCC from ICC. The regularization parameter (λ) of the selected features was adjusted with 10-fold cross-validation using the 1-standard-error (1-SE) method.

### 2.5. Model Establishment and Evaluation

The optimal radiomics features selected from each sequence were used to establish radiomics models (FS-T_2_WI, AP and PVP models), and clinical variables were used to establish the clinical model (C model) with logistic regression. By integrating the optimal radiomic features from each model, a joint radiomics model (JR model) was established [45]. Finally, the optimal radiomics features and clinical risk factors were combined to establish the radiomics–clinical model (RC model). The efficacy of the models was assessed considering the area under the receiver operating characteristic (ROC) curve (AUC), sensitivity, specificity, positive predictive value (PPV), negative predictive value (NPV), accuracy and F1 score as determined from the logistic regression confusion matrix.

### 2.6. Statistical Methods

R software (4.1.2, https://www.r-project.org/, accessed on 8 December 2021) was used for statistical data processing. Specifically, the software packages “psych”, “glmnet” and “pROC” were used to assess the intra- and intergroup consistency in the radiomics features to perform LASSO regression analysis and to plot the ROC curves, respectively. The normality and homogeneity of variance of the quantitative data were tested using the Shapiro–Wilk test and Bartlett test, respectively. The independent-samples *t* test was performed for quantitative data with a normal distribution and homogeneous variance; otherwise, the Mann–Whitney U test was performed. Quantitative data are presented as the means or medians. Categorical variables were analyzed using the chi-square test and are presented as percentages. Two-sided *p* values < 0.05 were considered to indicate statistical significance.

## 3. Results

### 3.1. Patient Characteristics

Among the 177 patients, 129 had HCC (112 men and 17 women) and 48 had ICC (19 men and 29 women). Cirrhosis occurred in 121 patients (107 with HCC and 14 with ICC), and multinodular liver cancer occurred in 65 patients (47 with HCC and 18 with ICC). The maximum tumor diameter was 6.57 ± 3.22 cm (Table 2). The HCC and ICC groups demonstrated significant differences in gender, serum AFP and extrahepatic metastasis status.

### 3.2. Feature Extraction and Selection

**Figure 3:**
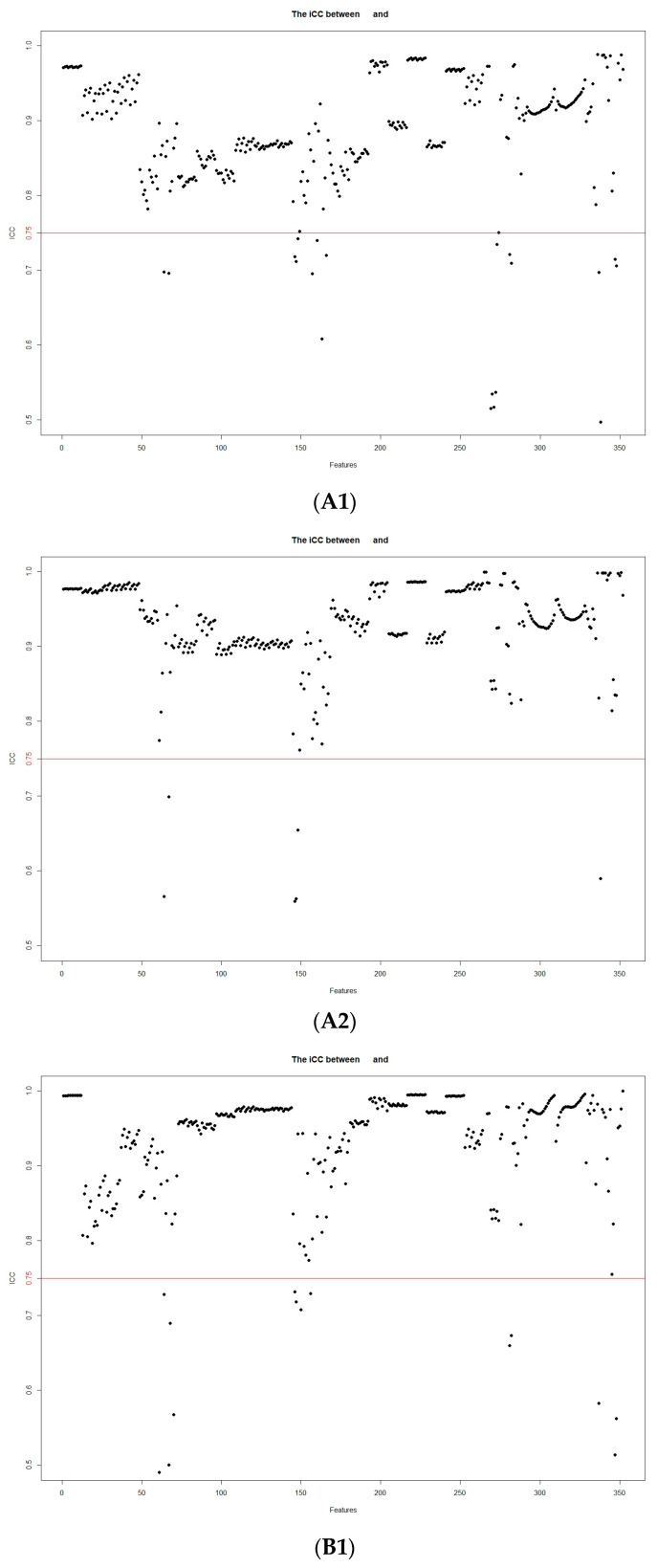

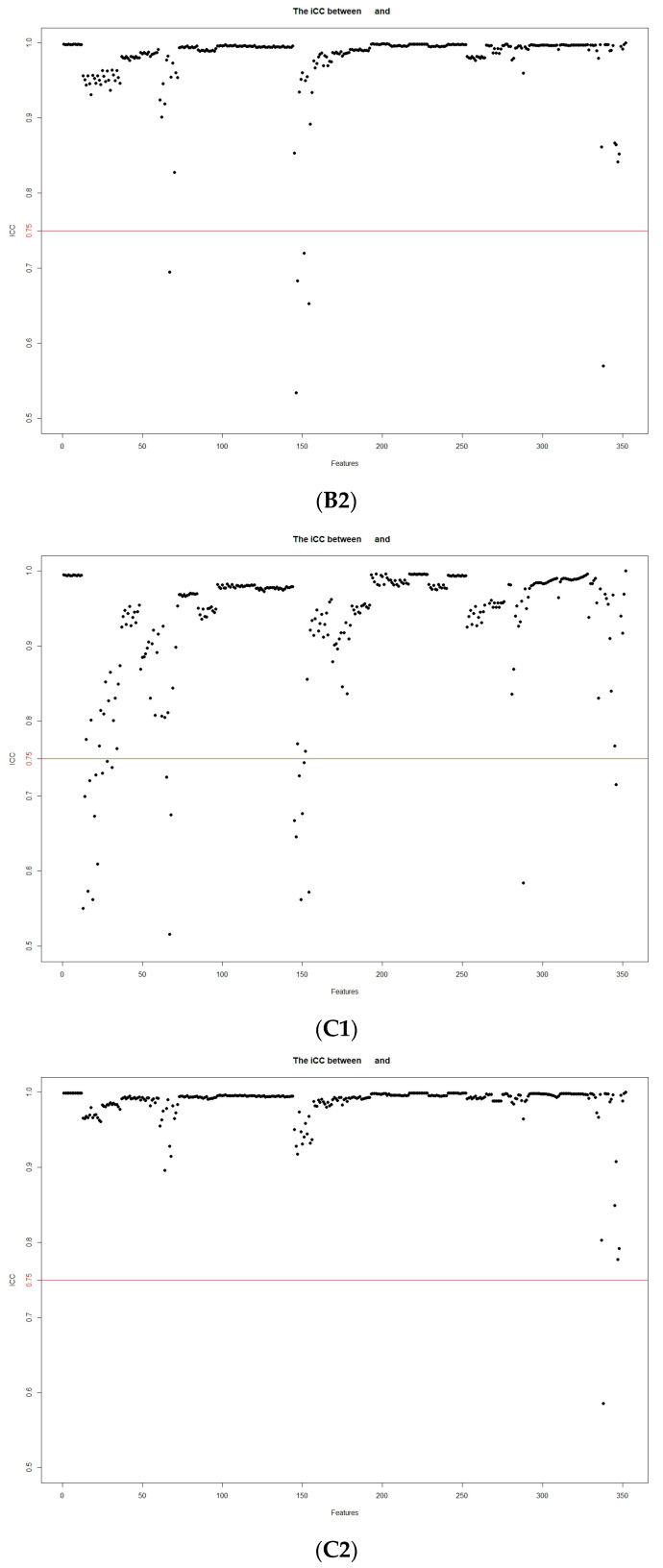
Stability assessment of the extracted MRI radiomic features according to inter- and intraobserver intraclass correlation coefficients: (**A1**) intergroup FS-T_2_WI; (**A2**) intragroup FS-T_2_WI; (**B1**) intergroup arterial phase; (**B2**) intragroup arterial phase; (**C1**) intergroup portal venous phase; (**C2**) intragroup portal venous phase.

A total of 352 features were extracted from each of the FS-T_2_WI, AP and PVP datasets. Features with intra- and intergroup intraclass correlation coefficients <0.75 were eliminated, and the remaining features were further analyzed (Figure 3).

**Figure 4:**
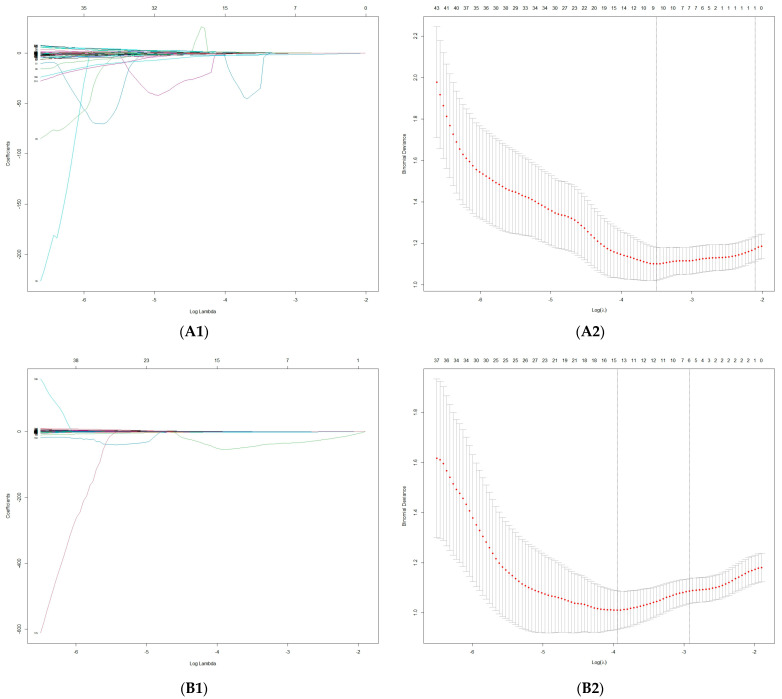

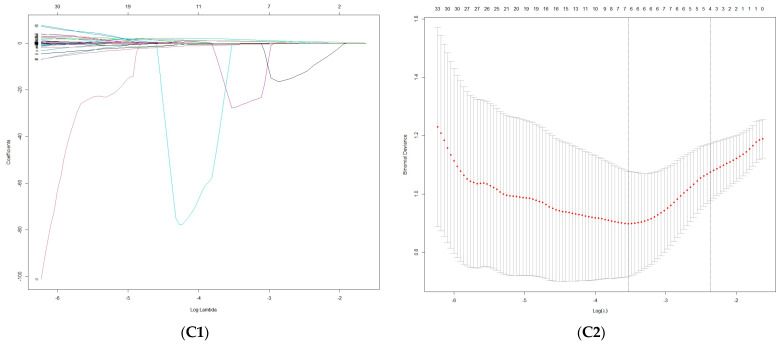
Feature selection using LASSO. (**A1**–**C1**) LASSO coefficient profiles of the radiomics features in the FS-T_2_WI, arterial phase and portal venous phase; (**A2**–**C2**) mean square error path using 10-fold cross-validation in the FS-T_2_WI, arterial phase and portal venous phase, respectively.

There were 327, 331 and 319 significantly different features in the FS-T_2_WI, AP and PVP datasets, respectively (*p* < 0.05), according to the independent-samples *t* test or Mann–Whitney U test. Finally, one, six and four optimal features, respectively, from these datasets were selected in LASSO regression (Figure 4 and Table 3).

### 3.3. Model Evaluation

**Figure 5:**
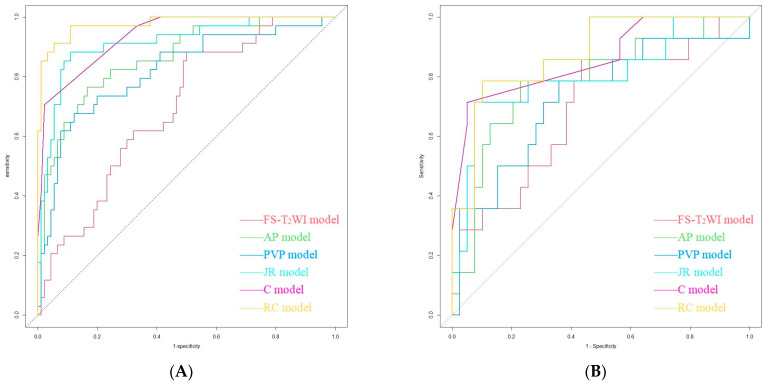
Performance of the FS-T_2_WI model, AP model, PVP model, JR model, C model and RC model in identifying HCC and ICC in the training group (**A**) and validation group (**B**) as detected using ROC curve analysis.

The AUCs of the FS-T_2_WI, AP, PVP, JR, C and RC models for distinguishing HCC from ICC were 0.693, 0.863, 0.818, 0.914, 0.936 and 0.977 in the training group and 0.690, 0.784, 0.727,0.802, 0.860 and 0.877 in the validation group, respectively (Table 4 and Figure 5).

## 4. Discussion

MRI is characterized by high soft-tissue contrast, multiparametric and multidirectional imaging and a lack of radiation, making it the preferred imaging method for identifying and diagnosing liver nodules [57,58]. Dynamic-contrast-enhanced MRI (DCE-MRI) is superior to dynamic-contrast-enhanced CT in the detection and diagnosis of small HCC lesions (maximum diameter ≤ 2.0 cm) [59,60]. Typical HCC displays significant heterogeneous enhancement in the arterial phase on DCE-MRI and reduced enhancement in the portal and/or parenchymal phase relative to that of normal liver parenchyma, resulting in a “fast-in and fast-out” enhancement pattern [25,28]. In contrast, ICC shows less obvious enhancement or mild heterogeneous enhancement in the arterial phase on DCE-MRI that gradually increases with time [26,27]. However, it is still difficult to differentiate HCC from ICC in clinical practice. Studies [30,34,35,36] have shown that small ICC lesions (diameter < 3 cm) and some ICC lesions in the setting of cirrhosis (approximately 7%) show the same enhancement pattern as typical HCC lesions, and approximately 10–20% of HCC lesions (especially small, hypovascular or sclerosing HCC lesions) show less obvious enhancement on imaging.

Choi et al. [61] conducted gadoxetic-acid-enhanced MR and dynamic CT scans to identify HCC and ICC. The results showed that PVP washout instead of conventional washout in gadoxetic-acid-enhanced MRI can prevent the misidentification of HCC as ICC in patients with cirrhosis; however, it reduces the sensitivity of the method for identifying HCC. Diffusion-weighted imaging (DWI) reflects the diffusion of water molecules in tissues by measuring the apparent diffusion coefficient (ADC). Wei et al. [62] and Lewis et al. [63] found that the ADC can help differentiate HCC from ICC. However, ICC has multiple cellular origins and shares similar biological behaviors with HCC to some extent; thus, the ADC of ICC can partially overlap with that of HCC. Additionally, DWI does not display small lesions well because of the limited spatial resolution, and conventional DWI is based on a monoexponential model that cannot differentiate between water molecule diffusion and blood perfusion [64]. Intravoxel incoherent motion-DWI (IVIM-DWI) can simultaneously quantify the diffusion of water molecules and microcirculatory perfusion in living tissues. A previous study [65] showed that both the ADC and D_slow_ values were significantly lower in HCC than in ICC, but the D_fast_ value was significantly higher in HCC than in ICC; furthermore, D_fast_ was more efficient in the differential diagnosis of HCC and ICC, and there was no significant difference in the f value between D_fast_ and D_slow_. The value of IVIM-DWI in identifying HCC and ICC has also been reported by other scholars [62,66,67]. However, the conclusion regarding D_fast_ and f in distinguishing HCC from ICC remains inconsistent or controversial; thus, further research is needed. As an effective tumor imaging tool, positron emission tomography (PET)-MRI can play a role in patient management. Çelebi et al. [68] argued that PET-MRI using ^18^F-fluorodeoxyglucos (^18^F-FDG) as a tracer agent can help differentiate between HCC and ICC. However, there is a need to deeply explore whether there are substantial differences in FDG uptake between HCC and ICC, the accuracy of identification in certain challenging cases (e.g., specific subgroups of patients in which the standard uptake value (SUV) is not a determining factor) and the optimal imaging sequence and model.

To date, few studies have investigated the differentiation of HCC from ICC based on MRI radiomics [42,44,63,69]. Liu et al. [44] adopted machine-learning-based CT and MR image features in the identification of cHCC-CC, ICC and HCC. The results showed that MRI features had the highest efficacy in differentiating between cHCC-CC and non-cHCC-CC, while CT features were less valuable. Moreover, precontrast- and portal-phase CT features were superior to enhanced MRI features in differentiating between HCC and non-HCC (AUC = 0.79–0.81 for MRI, 0.81 for precontrast-phase CT and 0.71 for portal-phase CT). Wang et al. [69] used MRI radiomics to preoperatively identify cHCC-CC, HCC and ICC and found that the performance of the higher-order feature-based model exceeded that of the lower-order feature-based model by approximately 10% and that the former performed better in identifying HCC in the delayed phase. Lewis et al. [63] extracted first-order radiomics features from ADC data and evaluated the ability of these features and the Liver Imaging Reporting and Data System (LI-RADS) classification to differentiate HCC, ICC and cHCC-CC. The results revealed that the AUCs of the combination of sex, LI-RADS grade and the fifth percentile of the ADC in diagnosing HCC were 0.90 and 0.89 for two independent observers, respectively. T_2_*WI can reflect the magnetic susceptibility variation in tissues and thus be used to assess the biological properties of tumor tissues [70]. Huang et al. [42] extracted radiomic features from T_2_*W images and then established radiomic nomogram models combined with clinical risk factors to distinguish between HCC and ICC. The results showed that the AUCs of the radiomics model were 0.90 and 0.91 in the training and validation groups, respectively, the AUCs of the clinical features were 0.88 in the training group and 0.83 in the validation group, and the AUCs of the radiomics nomogram were 0.97 and 0.95 in the training and validation groups, respectively. Similar results were obtained by Zhou et al. [43]. However, the efficacy of a joint model incorporating multiple sequence features was not investigated in these studies.

Different kinds of information related to tumor structure can be revealed by different sequences: T_2_WI exhibits the underlying tumor morphology and heterogeneity, and enhanced scans can reflect differences in the tumor blood supply. In this work, enhancements in the arterial and venous phases were combined based on T_2_WI to explore the efficacy of a joint model according to the blood supply status and enhancement patterns of HCC and ICC. The results showed that while each model had the potential to identify HCC and ICC in both the training and validation groups, the joint model incorporating multiple sequence features showed the highest efficacy [44,69]. Radiomics features based on MRI in combination with clinical risk factors are valuable for liver tumor differentiation [71]. In this study, univariate and multivariate analyses indicated that gender, serum AFP and extrahepatic metastasis status were independent clinical risk factors. The model integrating the radiomics features and clinical risk factors showed a further improvement in performance. The AUC of the T_2_WI model was relatively low in this study, consistent with the findings of Liu et al. [72]. Therefore, the value of FS-T_2_WI-based radiomics in distinguishing between HCC and ICC remains to be properly determined with further research.

In this study, we used the LASSO for feature selection. The LASSO is a well-known regularization technique which is popularly used in radiomics studies. One of the most unique advantages of this technique is that it reduces overfitting without limiting a subset of the dataset to only be used for internal validation. However, the LASSO does not eliminate the need to validate models in external datasets. A corresponding important disadvantage of the LASSO method is that the regression coefficients may not be reliably interpretable in terms of independent risk factors, as its focus is on the prediction of the optimal combination, rather than the accuracy of the estimation and interpretation of the contribution of individual variables [73]. The radiomics features selected were mainly GLCM and GLRLM features, textural features used to quantify tumor heterogeneity by reflecting the relationship between adjacent voxels/pixels [74], which is consistent with the results of several related studies [42,44,75,76,77,78,79,80]. Histogram features show the global distribution of grayscale values in the image and can also be used to assess tumor heterogeneity [81]. Lewis et al. [63] found that the 5th/10th/95th percentiles of the ADC could significantly differentiate HCC from ICC and cHCC-CC. Shape features reflect the geometric characteristics of tumors [74]; Zhao et al. [82] confirmed that HCC tends to be more spherical than ICC in terms of morphology.

This study had the following limitations. (1) In this retrospective study, many HCC and ICC patients who did not undergo pretherapeutic MRI scans were excluded, so there may be a potential selection bias. (2) The sample was small and from a single center, and cHCC-CC and ICC types other than the mass-forming type were not included in the study. In the future, the sample size should be expanded to multiple centers for further external validation. (3) Other relevant MRI sequences were not analyzed, so their potential contributions might have been ignored.

## 5. Conclusions

Multisequence MRI radiomics models can be used to pretherapeutically distinguish between HCC and ICC; the combined model integrating the optimal radiomic features with clinical risk factors can further improve the identification performance.

## Figures and Tables

**Table 1:** MRI sequence and parameters.

Sequence	TR/TE (ms)	FA (°)	Matrix (mm^2^)	FOV (mm^2^)	ST (mm)
BH Ax LAVA-Flex	4/2	12	260 × 192	320 × 320–360 × 360	2.6
RTr Ax fs T_2_WI	2609/97	110	384 × 384	320 × 320–380 × 380	5
BH Ax LAVA-Flex+C	4/2	12	224 × 192	320 × 320–360 × 360	5

Notes: TR, repetition time; TE, echo time; FA, flip angle; FOV, field of view; ST, section thickness; LAVA-Flex, liver acquisition with volume acceleration flexible.

**Table 2:** Patient clinical characteristics.

Parameter	Training Cohort (*n* = 124)	Validation Cohort (*n* = 53)	*p* Value
Sex			0.565
Male	94	38	
Female	30	15	
Age			0.751
≤60	78	32	
>60	46	21	
Satellite nodules			0.618
Yes	47	18	
No	77	35	
Diameter			0.076
≤5	41	25	
>5	83	28	
Ascites			0.514
Yes	36	18	
No	88	35	
Hemorrhagic necrosis			0.162
Yes	86	31	
No	38	22	
Pseudocapsule			0.975
Yes	26	11	
No	98	42	
Extrahepatic metastases			0.234
Yes	23	6	
No	101	47	
Portal vein tumor thrombus			0.445
Yes	35	18	
No	89	35	
Cirrhosis			0.533
Yes	83	38	
No	41	15	
Hepatitis B or C			0.891
Yes	90	39	
No	34	14	
AFP (ng/mL)			0.259
<20	54	30	
20~400	21	8	
>400	49	15	
DCP (mAU/mL)			0.905
≤27.8	11	5	
>27.8	113	48	
CA19-9 (U/mL)			0.244
≤37	68	24	
>37	56	29	
CEA (µg/L)			0.601
≤5	80	32	
>5	44	21	
Histologic result			0.891
HCC	90	39	
ICC	34	14	

Notes: HCC, hepatocellular carcinoma; ICC, intrahepatic cholangiocarcinoma; AFP, alpha-fetoprotein; CA19-9, carbohydrate antigen 19-9; DCP, des-gamma-carboxy prothrombin; CEA, carcinoembryonic antigen.

**Table 3:** Radiomics features identifying HCC and ICC selected from each dataset in LASSO regression.

Cohort	Feature Type	Feature Name
FS-T_2_WI	Shape features (*n* = 1)	Roundness
Arterial phase	Texture features (*n* = 3)	
	GLCM (*n* = 1)	45-7InverseDiffMomentNorm
	GLRLM (*n* = 2)	0LongRunEmphasis
		90ShortRunLowGrayLevelEmpha
	Intensity histogram features (*n* = 1)	InterQuartileRange
	Shape features (*n* = 2)	Mass
		Roundness
Portal venous phase	Texture features (n = 2)	
	GLCM (*n* = 2)	90-1Contrast
		45-7InverseDiffMomentNorm
	Intensity histogram features (*n* = 2)	InterQuartileRange
		MeanAbsoluteDeviation

Notes: HCC, hepatocellular carcinoma; ICC, intrahepatic cholangiocarcinoma; FS-T_2_WI, fat suppression T_2_-weighted imaging; GLCM, gray-level cooccurrence matrix; GLRLM, gray-level run-length matrix. GLCM features were constructed in four directions (θ = 0°, 45°, 90° and 135°) and three offsets (d = 1, 4 and 7); GLRLM features were constructed in two directions (θ = 0°, 90°) and one offset (d = 1).

**Table 4:** Efficacy of each model in identifying HCC and ICC.

Cohort	Model	AUC	Sen	Spe	PPV	NPV	ACC	F1 Score
Training	FS-T_2_WI model	0.693	0.147	0.956	0.556	0.748	0.734	0.233
	AP model	0.863	0.588	0.933	0.769	0.857	0.839	0.667
	PVP model	0.818	0.588	0.922	0.741	0.856	0.831	0.656
	JR model	0.914	0.706	0.922	0.774	0.892	0.863	0.738
	C model	0.936	0.706	0.978	0.923	0.898	0.903	0.800
	RC model	0.977	0.853	0.978	0.935	0.946	0.944	0.892
Validation	FS-T_2_WI model	0.690	0.071	0.974	0.5	0.745	0.736	0.125
	AP model	0.784	0.571	0.897	0.667	0.854	0.811	0.615
	PVP model	0.727	0.357	0.897	0.556	0.795	0.756	0.435
	JR model	0.802	0.571	0.923	0.727	0.857	0.83	0.640
	C model	0.860	0.714	0.949	0.833	0.902	0.887	0.769
	RC model	0.877	0.714	0.897	0.714	0.897	0.849	0.714

Notes: HCC, hepatocellular carcinoma; ICC, intrahepatic cholangiocarcinoma; FS-T_2_WI, fat suppression T_2_-weighted imaging; AP, arterial phase; PVP, portal venous phase; JR, joint radiomic; C, clinical; RC, radiomics–clinical; AUC, area under the receiver operating characteristic curve; ACC, accuracy; Sen, sensitivity; Spe, specificity; PPV, positive predictive value; NPV, negative predictive value.

## Data Availability

The data presented in this study are available upon request from the corresponding author.
